# Understanding the Exposed Area Dependent Photoelectrochemical Performance of BiVO_4_ Photoanodes

**DOI:** 10.1021/acsmeasuresciau.6c00248

**Published:** 2026-09-16

**Authors:** Christian Robles, Ana Gutiérrez-Blanco, Laura Montañés, Agustin O. Alvarez, Camilo A. Mesa, Francisco Fabregat-Santiago, Sixto Giménez

**Affiliations:** † Institute of Advanced Materials, 83151 Universitat Jaume I, Avinguda de Vicent Sos Baynat, s/n, 12006 Castelló de la Plana, Spain; ‡ AMOLF, Science Park 104, 1098 XG Amsterdam, The Netherlands

**Keywords:** bismuth vanadate, photoelectrochemistry, photoanodes, perfomance benchmarking, charge carrier dynamics, geometrical artifacts

## Abstract

To enable meaningful comparisons in solar fuel production, the energy conversion efficiency of photoelectrodes must be evaluated using standardized benchmarking protocols. For solar water splitting, the photocurrent density at a defined potential in a three-electrode configuration (e.g., 1.23 V vs. RHE for water oxidation) is widely used. However, this single descriptor is often insufficient for reliable cross-comparison between studies. In many reports, the highest photocurrent densities are obtained under conditions where the entire geometric electrode area (typically several cm^2^) is illuminated, while only a small fraction (<0.1 cm^2^) is in contact with the electrolyte. This discrepancy highlights the critical role of the fabricated or illuminated-to-exposed area ratio on the measured performance. In the present study, we systematically investigate the dependence of photocurrent density on the exposed-to-fabricated area ratio of bismuth vanadate (BiVO_4_) photoanodes prepared using state-of-the-art methods. By combining electrochemical, spectroscopic and structural characterization, we elucidate how this geometric factor governs the apparent photoelectrochemical response. Our results demonstrate that photocurrent metrics are highly sensitive to mismatches between illuminated, fabricated, and electrolyte-exposed areas, underscoring the need for more transparent and standardized reporting protocols to ensure meaningful benchmarking of photoelectrodes across studies.

## Graphical abstract

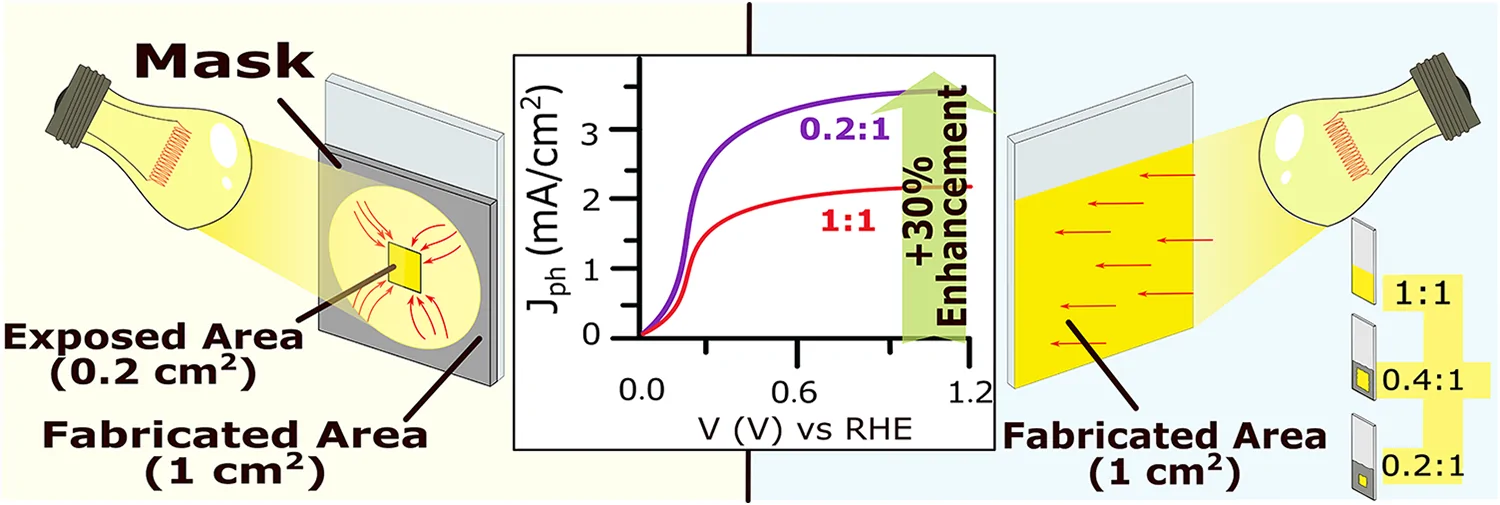

## 1 Introduction

The demand of green and sustainable energy sources is increasing rapidly to replace fossil fuels. Despite its intermittency, solar energy is by far the most promising renewable energy source currently available. For this reason, several energy conversion schemes have been developed to capture and store it. In particular, in the last decade, photoelectrochemical (PEC) processes such as water splitting, using Earth abundant metal oxide photoelectrodes (TiO_2_, BiVO_4_, Fe_2_O_3_, Cu_2_O, etc...) have attracted a great deal of attention to generate sustainable fuels (like green hydrogen) and added-value chemicals.^1,2^ However, the energy conversion efficiency of these photoelectrodes is generally low, due to excessive electron-hole recombination, slow charge transport and poor water oxidation kinetics.^3–5^ Benchmarking their performance is generally based on PEC characterization in a three-electrode cell, reporting the photocurrent density obtained at a certain electrochemical potential (e.g., 1.23 V vs. RHE for water oxidation photoanodes and 0 V vs. RHE for hydrogen evolving photocathodes).^6^ However, despite various efforts to establish unified benchmarking and characterization protocols,^7–10^ the PEC field still lacks consistent procedures comparable to those used in photovoltaics (PV) and batteries, particularly for measuring and reporting energy conversion efficiency and device stability.^6,11^

Bismuth vanadate (BiVO_4_) is a semiconductor material, widely studied for photoelectrochemical water oxidation due to its moderate bandgap (∼2.4 eV), which enables visible-light absorption, and its favorable valence band position for oxygen evolution.^12–16^ Reported photocurrent densities have exceeded 7 mA·cm^–2^,^17,18^ (93% of its theoretical photocurrent, 7.5 mA·cm^–2^)^5^ although this impressive performance strongly depends on the measurement conditions, particularly on the ratio of the exposed area of the photoactive material in contact with the electrolyte with respect to the fabricated area (referred as exposed-to-fabricated area ratio or XX:YY in the present work). In fact, the measured photocurrent density is systematically higher when the PEC response is evaluated at a low exposed-to-fabricated area ratio (see Figure 1). It is apparent that photocurrent densities are usually measured on both 1 cm^2^ or on smaller areas defined upon masking, being ∼0.2 cm^2^ a typical value found in the literature. This dependence of the photoelectrode *J*–*V* response on the masking (or exposed/illuminated ratio) has already been reported for dye sensitized solar cells (DSSCs) by Grätzel and co-workers, who demonstrated that the aperture size of the mask strongly influences the measured PV efficiency and proposed a reliable protocol for measuring small DSSCs.^19^ The size of the mask aperture critically affects the device performance by influencing the photocurrent density and the open-circuit voltage (*V*_OC_). From a semiconductor physics perspective, when the illuminated or fabricated area is larger than the electrolyte-exposed area, the shaded regions of the film act as a parallel dark diode. Photogenerated charge carriers can diffuse laterally into these unilluminated zones. This process significantly increases the overall diode reverse saturation current (*J*_0_) due to enhanced non-radiative recombination channels within the unexcited bulk or at the back contact, ultimately leading to a severe depletion of the steady-state *V*_OC_. Consequently, the *V*_OC_ decreases with decreasing aperture size, due to the enhanced contribution of dark current from the covered fraction of the TiO_2_ skeleton. While in photoelectrochemical systems like BiVO_4_ the macroscopic catalytic dark current onset for water oxidation is typically located hundreds of millivolts away from the OCP, this junction-related recombination artifact highlights how geometric mismatches fundamentally skew the electronic equilibria of the electrode.

**Figure 1:**
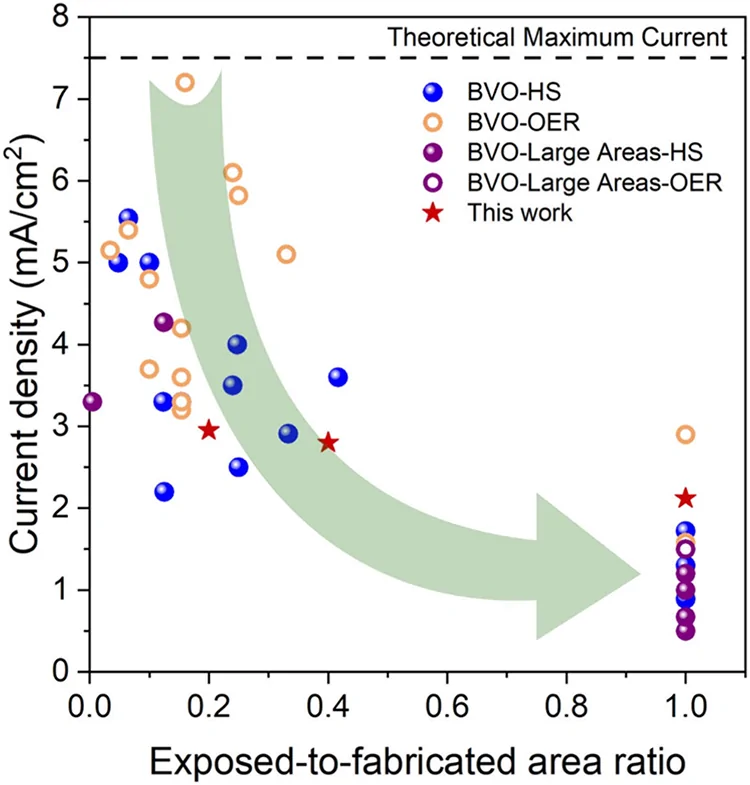
Photocurrent density (normalized to the exposed area) at 1.23 V vs. RHE as a function of the exposed-to-fabricated area ratio for different BiVO_4_ photoanodes reported in the literature (Supporting Information, Table S1). Small-scale samples (blue/orange circles) are compared against large-area devices (purple circles). Filled symbols represent measurements performed using a hole scavenger (HS), while open symbols represent measurements for the Oxygen Evolution Reaction (OER) performed in the presence of a co-catalyst. The results of the present study are showed as red stars.

More recently, Kim et al. systematically investigated this phenomenon in BiVO_4_ photoanodes for water splitting, providing a detailed analysis of how optical artifacts affect PEC measurements.^20^ They showed that backscattered and internally reflected light within the masked region contributes significantly to the apparent photocurrent, particularly under conditions where the illuminated area exceeds the active electrode–electrolyte interface. This results in an artificial enhancement of photocurrent density, which does not reflect the intrinsic activity of the material, but rather optical redistribution effects. Importantly, their study emphasized that the magnitude of this artifact increases with electrode roughness and thickness, making direct comparison between differently prepared electrodes unreliable, unless masking conditions are standardized. Together, these findings highlight the critical need to carefully control and report illumination geometry to avoid overestimation of PEC performance.

Related effects have also been considered in the context of large-area BiVO_4_ photoanodes and electrode architecture. Yao et al. reported the so-called “areal effect”, showing a systematic decrease in photocurrent density with increasing illuminated area, and suggested electrical resistance of the fluorine-doped tin oxide (FTO) substrate and ionic diffusion as possible contributors, while leaving its fundamental origin unresolved.^21^ Ahmet et al. demonstrated a 50 cm^2^ BiVO_4_ photoanode for tandem PEC–PV water splitting, reporting a decrease in photocurrent density from 3.3 to 1.2 mA·cm^–2^ when increasing the photoanode area from 0.24 to 50 cm^2^; the incorporation of Ni conductive lines partially mitigated this loss.^22^ Ju et al. showed that patterned FTO substrates and large-area electrode architectures can enhance PEC performance through improved light management and increased effective surface area,^23^ whereas Murugan et al. attributed the photocurrent losses observed upon scaling BiVO_4_ photoanodes from 1 to 25 cm^2^ primarily to increased ohmic losses associated with the FTO substrate.^24^

These studies establish that electrode area and architecture are important parameters in BiVO_4_ PEC measurements, but they do not address the effect of decoupling the fabricated, illuminated and electrolyte-exposed areas. This is particularly relevant because photogenerated carriers may originate from regions that are not directly exposed to the electrolyte, while the measured photocurrent density is commonly normalized to the exposed area. In the present study, we therefore maintain a constant fabricated and illuminated area while systematically vary the electrolyte-exposed area. By combining electrochemical impedance spectroscopy (IS), intensity-modulated photocurrent spectroscopy (IMPS) and intensity-modulated photovoltage spectroscopy (IMVS), we investigate how this exposed-to-fabricated area mismatch affects the apparent PEC response and the extraction of charge transport/transfer parameters. Our results demonstrate that the resulting apparent performance enhancement is predominantly geometrical rather than an intrinsic improvement of the BiVO_4_ photoelectrode, highlighting the need to distinguish between illuminated, fabricated and electrolyte-exposed areas when benchmarking PEC performance.

## 2 Experimental Section

### 2.1 Synthesis of BiVO_4_ Photoanodes

BiVO_4_ photoanodes were prepared by a modified previously reported method.^25^ Before the film deposition, fluorine-doped tin oxide (FTO) substrates were washed ultrasonically in soap water (Extran, Sigma-Aldrich), Milli-Q water and in a mixture of acetone/isopropanol (1:3) each of 15 min. Subsequently, rinsed FTO substrates were cleaned using a UV ozone chamber right before their use. Firstly, the plating solution is prepared by adding 0.02 mol of Bi­(NO_3_)_3_ in 50 mL of a 0.4 M KI solution containing 0.06 mol of lactic acid at a pH 1.7 adjusted by HNO_3_. Then, 20 mL of a 0.23 M *p-*benzoquinone solution were slowly added into the plating solution under stirring. A three-electrode system was used for electrodeposition. A platinum sheet as counter electrode (CE), a Ag/AgCl (3 M KCl) as reference electrode (RE) and a FTO as working electrode (WE) were used. The electrochemical deposition was performed in two steps. First a potential of −0.35 V vs Ag/AgCl during 20 s was applied for the nucleation step. The growing step was performed by applying −0.1 V for 300 s at room temperature, which was equivalent to passing a charge of −0.19 C·cm^–2^. The precipitation of BiOI onto FTO was produced by the increase in local pH on the WE due to the reduction of *p-*benzoquinone to hydroquinone by applying a cathodic bias. The BiOI films were washed with milli-Q water and air-dried. Onto the electrodeposited BiOI films, 50 μL of 0.2 M VO (acac)_2_ solution in DMSO were deposited by drop-casting on a heating plate at 80 °C. The deposited films were calcinated at 475 °C (heating rate, 2 °C·min^–1^) for 1 h. The excess of V_2_O_5_ was removed by soaking the photoanodes in a 1 M NaOH solution under vigorous stirring for 10 min, followed by washing these films with milli-Q water and air-drying. The as prepared photoanodes were masked with an opaque epoxy resin to exposed areas of 1 cm^2^, 0.4 cm^2^ and 0.2 cm^2^ . High-quality digital images of the different masked samples were taken, and the “*ImageJ”* software^26^ with one pixel resolution (version 1.54d, NIH, Bethesda, MD, USA), was used to accurately determine the exposed area for each prepared photoanode, and consequently, to calculate precise photocurrent densities.

### 2.2 Physical Characterization

The surface morphology and composition of the samples were examined by field-emission scanning electron microscopy (FE-SEM) with a JSM-700F JEOL FEG-SEM system (Tokyo, Japan). The elemental analysis was performed by an INCA 400 Oxford EDS analyzer (Oxford, U.K.) operating at 15 kV. Before the FE-SEM experiment, the samples were sputtered with a 2 nm thick layer of Pt. Raman spectra were measured with a WITec Apyron confocal microscope using a 532 nm laser with a 1 mW power, a grating of 1800 g/mm, a BLZ = 500 nm and an optical objective Zeiss EC Epiplan-Neofluar Dic 50x/0.55. UV–vis absorption spectra were obtained on a Lambda 1050+ spectrophotometer (Perkin Elmer, Waltham, MA, USA) with an integrating sphere using BaSO_4_ as reference. The absorbance (*A*) was estimated by *A* = log­(*T* *+* *R*), where *T* is transmittance and *R* is diffuse reflectance. The indirect optical bandgap could be estimated by the Tauc plot as it is shown in the eq 1, where *E*_g_ is the transition energy, *h*ν is the photon energy and *A* is the proportionality constant.

1 $${\left(h\nu \alpha \right)}^{1/2}=Α\left(h\nu -E_{g}\right)$$

### 2.3 Photoelectrochemical Measurements

The photoelectrochemical (PEC) performance of the photoelectrodes was assessed using an Autolab potentiostat/galvanostat PGSTAT302, and the light source was a 300 W Xe lamp with AM 1.5 G, adjusting the light intensity to 100 mW·cm^–2^ using a Si photodiode. For the evaluation of the photoelectrodes activity, *J*–*V* curves with a scan rate of 5 mV·s^–1^ in the dark and under illumination were carried out at room temperature in a three-electrode cell with the photoanode, Pt coil and Ag/AgCl as a working, counter and reference electrodes, respectively. The electrolyte was a 0.1 M potassium phosphate (KPi) buffer aqueous solution (pH 7.0) with Na_2_SO_3_ serving as a sacrificial hole scavenger. Since the potential was measured against Ag/AgCl reference electrode, all the potential measurements were converted to the reversible hydrogen electrode (RHE) by the Nernst equation:

2 $$E_{\mathrm{RHE}}=E_{\mathrm{Ag}/\mathrm{AgCl}}+E_{\mathrm{Ag}/\mathrm{AgCl}}\left(\mathrm{reference}\right)+0.0591\;V\times \mathrm{pH}$$

3 (EAg/AgCl(reference)=0.1976VvsNHEat25C◦)

Chronoamperometry (CA) measurements under infrared (IR) illumination were performed using a mobile Palmsens3 potentiostat (PalmSens BV, Houten, Netherlands). For these measurements, identical experimental conditions as those described above were employed. An infrared diode laser (MDLIII-980-2W, Roithnere LaserTechnik, Vienna, Austria,980 nm ± 5 nm, 2 W cw, stability <5%, beam aperture of 5 × 8 mm^2^) was employed to illuminate the BiVO_4_ photoanodes, focusing the beam as close as possible to the photoanode. Chopped-light measurements were performed manually, carefully controlling the on/off timing to ensure consistent illumination cycles during the photoelectrochemical experiments

The JVs, CAs, and impedance spectroscopy (IS), measurements were carried out using an Autolab PGSTAT302 with a FRA32M module combined with a 300 W Xe lamp with AM 1.5 G and intensity modulated photocurrent spectroscopy (IMPS) and intensity modulated photovoltage spectroscopy (IMVS) with a Light-emitting diode (LED) driver; The LED intensity was 89 mW·cm^2^ generated by an array of three blue LEDs (Philips LUMILEDS LXML-PB01-0040 with 470 nm peak) applying an AC perturbation equal to 10% of the light flux. For the IS measurement, a 20 mV AC perturbation was applied. The frequency range was from 5 kHz to 1 Hz for the three techniques. The combination of IS, IMPS and IMVS was performed at a voltage of 1.23 V vs RHE. The analysis of all spectra was performed using MultiNonlinearModelFit function of Wolfram Mathematica software. For the IS, IMPS, IMVS spectra, they were simultaneously fitted using a unified transport–recombination model, as described in detail in our previous work.^27^ The model is based on the one-dimensional continuity equation for the carrier density *n*(*x*,*t*) in a device with spatially distributed photogeneration *G*(*x*,*t*), governed by a diffusion coefficient *D* and a recombination lifetime *τ*_rec_

4 $$\frac{\partial n\left(x,t\right)}{\partial t}=D\frac{\partial^{2}n\left(x,t\right)}{\partial x^{2}}-\frac{n\left(x,t\right)}{\tau_{\mathrm{rec}}}+G\left(x,t\right)$$

Analytical transfer functions were derived from this equation by applying the appropriate boundary conditions for each technique (EIS, IMPS, and IMVS), enabling the simultaneous fitting of the three frequency-domain responses using a common set of transport and recombination parameters.

## 3 Results and Discussion

Nanoporous BiVO_4_ photoanodes were fabricated by a previously reported electrodeposition method with minor modifications (Supporting Information, Figure S1). To control the exposed area, the 1 cm^2^ fabricated films were partially masked with an opaque epoxy resin (Supporting Information, Figure S2) to areas of 0.2 and 0.4 cm^2^ (0.2:1 and 0.4:1 samples); morphology, thickness and the uniformity and full coverage of the masking layer were verified by Scanning Electron Microscopy (Supporting Information, Figure S3a,b). During photoelectrochemical measurements, illumination was delivered through a 1 cm^2^ window in a black cardboard cover, ensuring a constant illuminated area regardless of the exposed area in contact with the electrolyte, aiming at minimizing the effect of the light scattering towards the PEC cell (Supporting Information, Figure S4a). Preliminary PEC characterization was conducted under both front and back side illumination (Supporting Information, Figure S5), showing that the best performance was achieved under back side illumination, in good agreement with previous reports.^5,29–31^ All subsequent measurements were performed under back side illumination.

The optical absorbance and external quantum efficiency of representative photoelectrodes are reported as Supporting Information, Figure S6. Although these photoanodes are usually tested for water oxidation, here we employed Na_2_SO_3_ 0.1 M as a sacrificial hole scavenger in potassium phosphate buffer 0.1 M (KPi), ensuring reproducible measurements by suppressing surface recombination losses and avoiding the need for cocatalyst deposition. The electrochemical cell used in these experiments is showed as Supporting Information, Figure S4a.

Figure 2a shows the *J*–*V* curves under illumination for 1 cm^2^ synthesized BiVO_4_ samples, with exposed areas of 0.2, 0.4, and 1 cm^2^, (0.2:1, 0.4:1 and 1:1 samples). To provide statistical significance to the analysis, four identical films were measured at each condition, and the average photocurrent density is reported together with its standard deviation (shaded area). The highest photocurrent density was obtained for the 0.2:1 sample, reaching 2.92 mA·cm^–2^ at 1.23 V vs RHE, which corresponds to a 30% enhancement, compared to the reference 1:1 sample, where the exposed area equals the fabricated one (1 cm^2^). However, when the exposed-to-fabricated area ratio is 1, the measured current density is independent of the active area, either for 0.2:0.2 or 1:1 samples (Figure 2b). All the tested samples exhibit a reasonable stability, without significant loss of activity after more than 2 h chronoamperometric experiment (Figure 2c), which is crucial for the validity of the electrical spectroscopic analysis detailed below.

**Figure 2:**
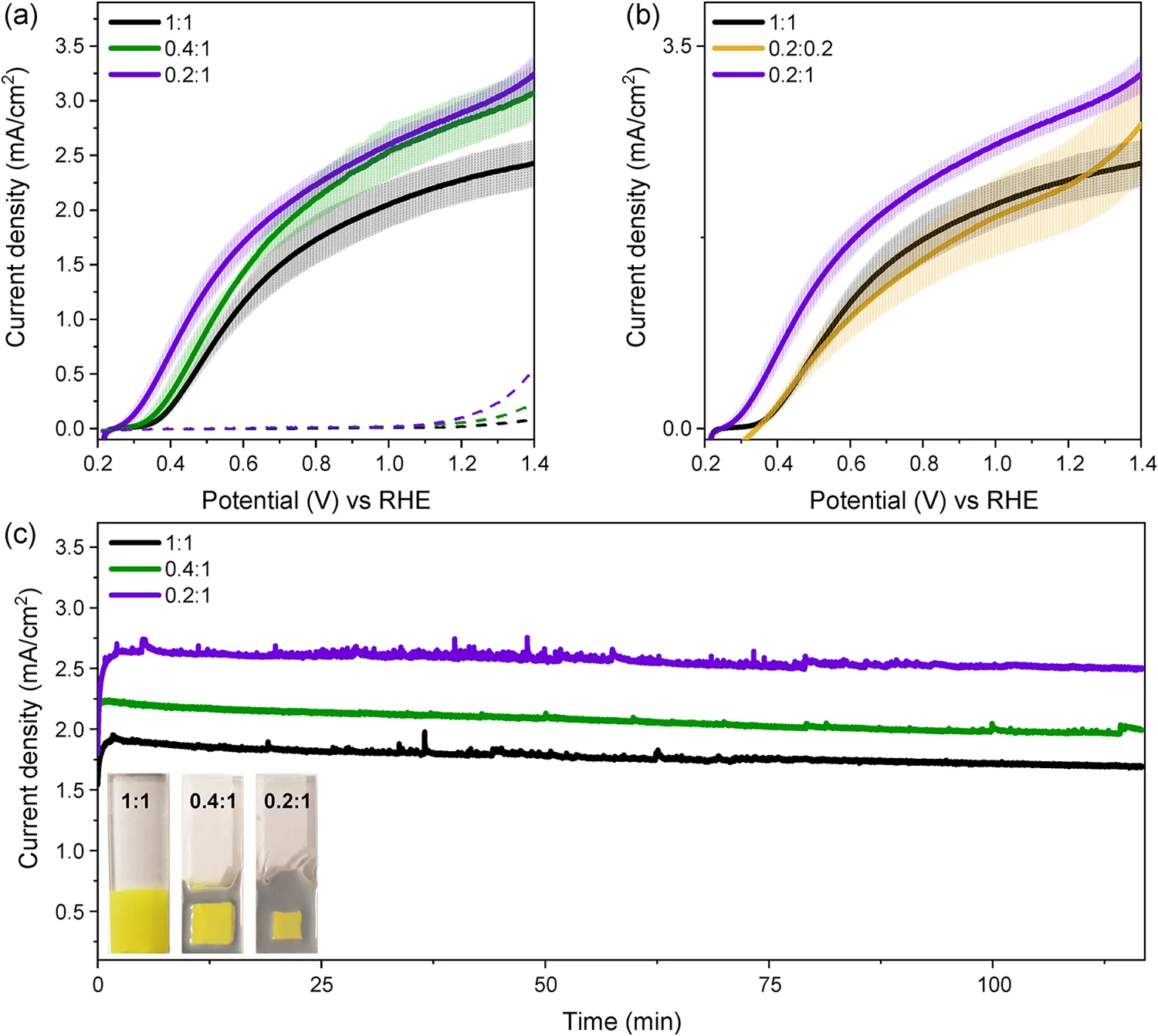
(a) Average photocurrent–voltage (*J*–*V*) plots for the BiVO_4_ photoanodes with different exposed-to fabricated area ratios, with a constant illumination area of 1 cm^2^. Dark currents are represented as dashed lines. (b) Average of *J*–*V* plots for an exposed and synthesized area of 1 cm^2^ (1:1 black line), an exposed and synthesized area of 0.2 cm^2^ (0.2:0.2 orange line) and an exposed area of 0.2 cm^2^ but a synthesized area of 1 cm^2^ (0.2:1 purple line), (c) chronoamperometric plots taken at 1.23 eV vs. RHE with an illumination area of 1 cm^2^. Inset: Image of the tested BiVO_4_ photoanodes with the exposed area delimited by Epoxy resin. All these measurements were carried out under back illumination in a 0.1 M potassium phosphate buffer solution (KPi) pH 7, with 0.1 M Na_2_SO_3_ as hole scavenger, under simulated sunlight (Xe source, AM 1.5G filter, 100 mW·cm^–2^).

Dark currents were also evaluated (Figure 2a) and showed to inversely scale with the exposed-to-fabricated area ratio. Representation of these dark curves without area normalization is showed as Supporting Information, Figure S7. Remarkably, all dark curves are identical within experimental error, suggesting that dark currents are related to FTO/electrolyte interface, which is independent of the exposed area of the electrode.

In order to rationalize the origin of this exposed-to-fabricated area ratio dependent behavior, photoelectrochemical impedance spectroscopy (IS), intensity modulated photocurrent spectroscopy (IMPS) and intensity modulated photovoltage spectroscopy (IMVS) measurements were carried out on the samples showed in Figure 2a,c. All the Nyquist plots from IS showed a single semicircle (Supporting Information, Figure S4b), which was systematically fitted to a simple *R*_*s*_ *-RC* circuit, where *R*_*s*_ is the series resistance, R combines the transport and charge transfer resistance (*R*_tr_ + *R*_ct_), being *R*_ct_ the main contribution, and C is the capacitance.^32^ Both *R* and *C* are reported as a function of applied voltage in Figure 3a,b, respectively. Upon normalization to the exposed area, *R* ≈ *R*_ct_ appears to scale with this exposed area, apparently suggesting a faster oxidation kinetics when this area is reduced. However, we believe that this apparent enhancement in PEC performance does not reflect the intrinsic photoelectrocatalytic activity of the material, most likely due to the contribution of photogenerated charges from absorbed photons outside the electrolyte-exposed area, as discussed below. This is also consistent with the light management issues described in previous studies,^22,23^ leading to a higher density of photogenerated charges participating per unit of normalized area, also in agreement with the *J*–*V* curves shown in Figure 2a.

**Figure 3:**
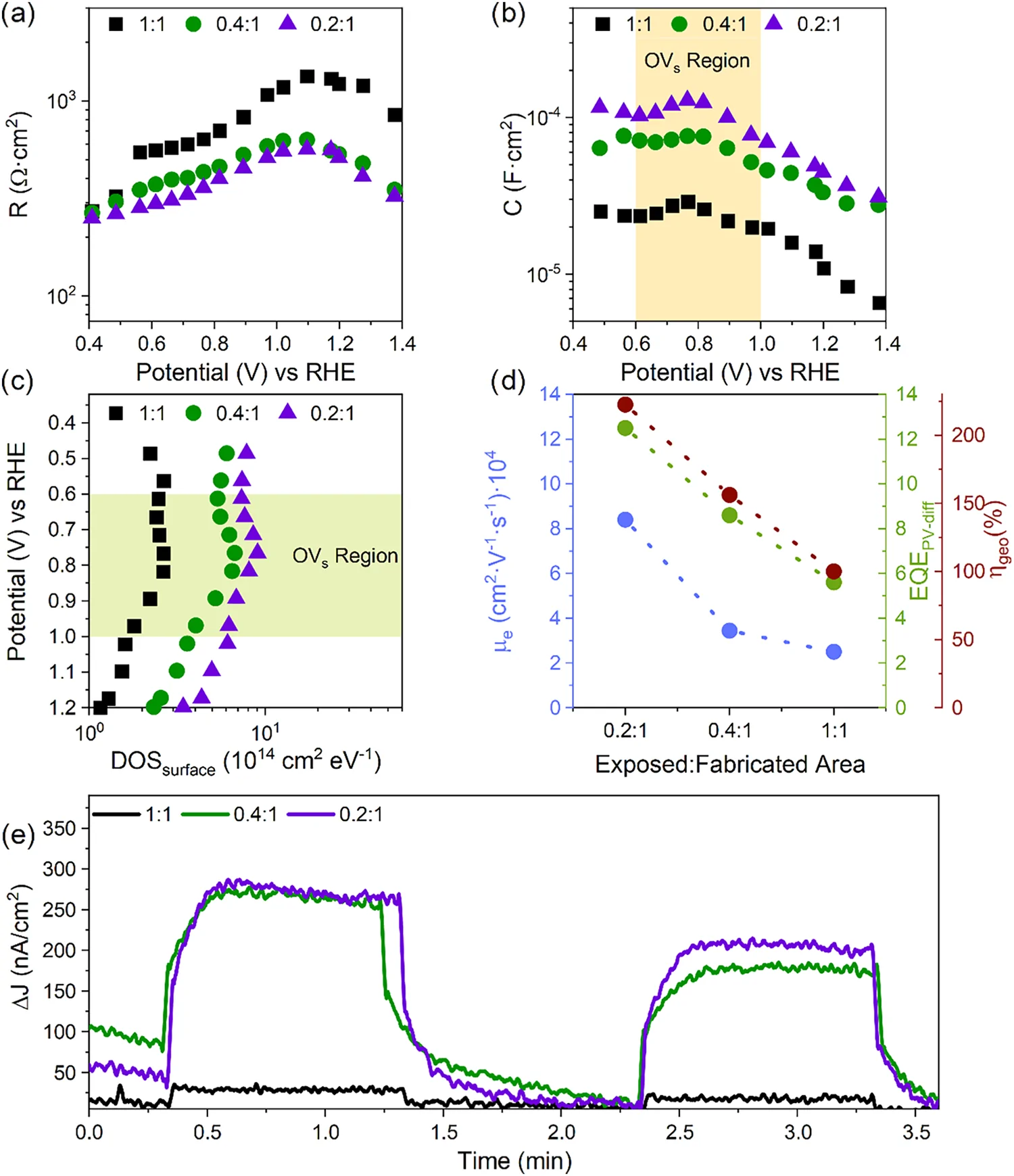
(a) Resistance (*R*), (b) Capacitance (*C*), (c) density of surface states (DOS_surface_) as a function of applied potential extracted from IS, (d) Electron mobility (μ_e_, blue), differential external quantum efficiency (EQE_PV‑diff_) (green) and collection efficiency (η_col_ %) (red) as a function of the exposed-to-fabricated area extracted from the IS, IMPS and IMVS analysis. The data is connected with dotted lines as an eye-guide. (e) IR-chopped chronoamperometry measurements at 1.23 V vs RHE (980 nm, 1.26 eV, 2 W).

On the other hand, the capacitance, *C* exhibits the characteristic exponential increase with applied potential expected for a chemical capacitance in all tested samples,^33^ with a pronounced maximum at 0.75 V vs RHE, which has previously been assigned to the V^4+^/V^5+^ redox transition and the concomitant formation of oxygen vacancies, (OV) (Figure 3b).^34,35^ Furthermore, the density of states at the surface (DOS_surface_) can be determined from eq 5

5 $$C=q\cdot {DOS}_{surface}$$

where *q* is the elementary charge (1.6·10^–19^ C).

Both the capacitance and the calculated DOS_surface_ appear to inversely scale with the exposed-to-fabricated area ratio (Figure 3b,c), suggesting an apparent increase in the density of surface defect states as the exposed-to-fabricated area ratio is reduced. As previously reported, these surface defect states exhibit photoactivity under infrared (IR) radiation.^36^ To further investigate this, complementary chronoamperometric measurements were performed under chopped infrared illumination (980 nm, 1.26 eV, 2 W). In line with previous findings, our results reveal a higher apparent sub-bandgap photoactivity for the 0.2:1 and 0.4:1 samples (Figure 3e), showing good agreement with the observed photocurrent (Figure 2a) and capacitance (Figure 3b) trends. A more detailed analysis indicates that the proportionality between the IR photocurrent and the extracted DOS_surface_ is roughly preserved in this case. While the DOS_surface_ increases only moderately, the IR photocurrent rises by nearly one order of magnitude as the exposed-to-fabricated area ratio decreases. These results suggest that the apparent enhancement in sub-bandgap photoactivity should be interpreted with caution and warrant further investigation to fully understand the underlying mechanisms.

While IS provides valuable insight into charge-transfer processes and the density of surface states, IMPS has been widely employed to probe surface recombination and interfacial charge-transfer kinetics in metal oxide photoelectrodes.^37–41^ When complemented by IMVS, the analysis becomes more robust, as photovoltage dynamics enable a clearer decoupling of bulk transport, recombination, and charge-transfer pathways.^27,42^ On this basis, an integrated IS/IMPS/IMVS methodology has emerged as a comprehensive framework for investigating charge-carrier dynamics in photovoltaic (PV) and photoelectrochemical (PEC) devices.^43–46^ Here, we simultaneously analyze the IS, IMPS, and IMVS responses of BiVO_4_ electrodes with different exposed-to-fabricated area ratios (Figures 2 and 3) at 1.23 V vs. RHE, following the methodology developed in our previous work.^27^ The model is summarized in the Experimental Section Supporting Information. The analysis has been carried out in front and back illumination, exhibiting consistent trends under both illumination configurations (see Tables S2 and S3). For the sake of clarity, here we will only focus on back illumination conditions.

The most relevant results of this analysis are summarized in Table 1 and Figure 3d. Figure 3d shows electron mobility (μ_e_), differential external quantum efficiency (EQE_PV–Diff_) and geometrical efficiency (η_geo_) as a function of the exposed-to-fabricated area ratio. Interestingly, a noticeable decrease in electron mobility (μ_e_) was observed as the ratio of exposed-to-fabricated area ratio increased. Peak mobility values (>8·10^4^ cm^2^ ·V^–1^· s^–1^) were achieved at a reduced exposed area (0.2:1 ratio), whereas scaling towards full surface contact (1:1 ratio) resulted in a substantial decrease of electronic transport properties, again suggesting an area-dependent transport limitation.

**Table 1:** Parameters Extracted from the Simultaneous Fitting of The IS, IMPS and IMVS spectra under Back Illumination

Sample code	1:1	0.4:1	0.2:1
EQE_PV‑diff_ [%]	5.6	8.6	12.5
*a* [%]	98.2	98.2	98.2
η_opt_ [%]	75.1	75.1	75.1
IQE_PV‑diff_ [%]	7.6	11.7	16.9
η_sep_ [%]	8	8	8
η_col_ [%]	95	94	95
η_geo_ [%]	100	156.2	222.5
η_sep_′ [%]	8	12.5	17.8

Similarly, the differential external quantum efficiency (EQE_PV–Diff_), which quantifies the number of electrons extracted per incident photon, also decreased as the ratio of exposed-to-fabricated area ratio increased. However, this decrease is significantly smaller compared to that for electron mobility, suggesting that charge transport kinetics are substantially more sensitive to the electrode–electrolyte interfacial geometry than the overall photon-to-electron conversion efficiency. While EQE_PV–Diff_ decreased by a factor of ∼2.2 when transitioning from a 0.2:1 to a 1:1 exposed-to-fabricated area ratio, the calculated electron mobility exhibited a much more pronounced decrease (factor 4). This sensitivity implies that the interface between the active layer and the electrolyte may introduce significant area-dependent transport limitations, such as increased surface recombination or interfacial resistance, as the effective interaction area increases.

A more detailed assessment of the EQE_PV–Diff_ efficiency can be carried out by considering the different factors contributing to this efficiency, expressed as:^27^

$${EQE}_{PV‐Diff}=\eta_{opt}a\eta_{sep}\eta_{col}\eta_{geo}$$ 6

Here the optical efficiency (η_opt_) accounts for photons reaching BiVO_4_ after reflections and other optical losses, which is independent of the exposed area (Table 1).^27^ Likewise, the probability of generating an electron-hole pair, given by the absorbance (*a*) does not vary with area. Combination of these terms defines the differential internal quantum efficiency *(*IQE_PV–Diff_ *):*

7 $${IQE}_{PV‐Diff}=\frac{{EQE}_{PV‐Diff}}{\eta_{opt}a}$$

which follows the same area-dependent trend as EQE_PV–Diff_ (Table 1). After photon absorption, the separation efficiency (η_sep_) determines the fraction of electron-hole pairs converted into free charges, while the collection efficiency (η_col_) reflects the fraction successfully extracted.

Since all measurements analyzed in Table 1 correspond to the same BiVO_4_ material, the separation efficiency, (η_sep_), is expected to be identical across all studied samples. However, when following the procedure from our previous work, where the geometrical efficiency (η_geo_) was not included in eq 6, different values of the separation efficiency, here defined as η_sep_^′^ (= η_sep_·η_geo_), were obtained for different exposed-to-fabricated area ratios. To account for these discrepancies, we introduced a geometrical efficiency (η_geo_) term to capture deviations from the reference 1:1 sample, for which η_geo_ is defined as 100%. As shown in Figure 3d, η_geo_ increases significantly as the exposed-to-fabricated area ratio decreases. In contrast the collection efficiency η_col_ remains nearly constant and close to 100% across all ratios, which is consistent with previous BiVO_4_ studies,^27^ confirming that η_sep_ is the rate-limiting step and responsible for the low EQE_PV–Diff_.

This integrated framework, supported by the quantitative parameters extracted from our combined IS/IMPS/IMVS analysis (Supporting Information, Tables S2 and S3) clarifies that the performance enhancements observed in Figure 2 are mirrored by the increase in EQE_PV–Diff_. Importantly, our analysis indicates that these improvements do not stem from intrinsic material or device properties, but rather from a geometrical artifact, most likely the additional contribution of photogenerated charges originating from photons absorbed outside the electrolyte-exposed region.

To further verify that intrinsic material changes do not contribute to the observed enhancement, we performed structural characterization of the BiVO_4_ films by Raman spectroscopy. It has been reported that prolonged illumination (photocharging) can generate oxygen vacancies (OV) via the V^4+^/V^5+^ redox process, which act as catalytically active sites and can boost PEC performance.^34,47,48^ However, our Raman analysis revealed no detectable changes in the A_g_ modes of V–O bonding, previously associated with OV formation (Supporting Information, Figure S8).^36,49^ These results confirm that the improved PEC response is not due to structural modifications, reinforcing the conclusion that the enhancement arises from extrinsic, geometry-related effects.

## 4 Conclusions

In conclusion, we have systematically investigated the influence of the exposed-to-fabricated area ratio on the photoelectrochemical performance of BiVO_4_ photoanodes fabricated by electrodeposition. Apparent performance increase upon decrease of this ratio was systematically observed. By combining IS, IMPS, and IMVS analysis, we demonstrated that the apparent enhancements in photocurrent density and differential quantum efficiency for smaller exposed-to-fabricated area ratios (0.2:1 and 0.4:1 samples) are primarily due to geometrical and optical effects rather than intrinsic improvements in the material or device properties, thus corrupting the diagnosis of intrinsic transport properties, and leading to misleading superior charge carrier dynamics that are by fact, geometric artifact.

While localized electronic measurements indicate subtle variations in the density of states (*DOS*), structural characterization by Raman spectroscopy confirmed that the bulk lattice integrity remains largely unaffected. This suggests that the observed macroscopic trends originate primarily from optical and geometrical artifacts such as photogenerated charges from regions outside the electrolyte-exposed area rather than being dominated by changes in defect density. These findings highlight the critical importance of standardizing measurement protocols, including the careful definition of exposed and illuminated areas, to ensure accurate and reproducible benchmarking of PEC photoelectrodes.

Given the significant changes in the estimated electron mobility as the exposed-to-fabricated area ratio scales up, our findings underscore a critical challenge in device characterization. To ensure broader comparability and practical relevance, we provide some recommendations:

- Recommended reporting of the exposed-to-fabricated area ratio: Authors should explicitly report not only the active area in contact with the electrolyte (*A*_exposed_) but also the total illuminated or fabricated area (A_illuminated_ or *A*_fabricated_). Reporting single photocurrent density metrics (mA·cm^−2^) without specifying this geometric ratio makes cross-laboratory replication nearly impossible.
- Digital Area Verification: As a standard practice, especially for small or irregular geometries where manual masking (e.g., epoxy resin or O-rings) introduces high tolerances, high-resolution digital photographs of the final electrode should be analyzed using pixel-counting software. This eliminates nominal area calculation errors.
- Establishing a Standard Baseline Configuration (1 cm^2^): We suggest that the community adopts a standard benchmarking baseline where *A*_exposed_ = *A*_illuminated_ = *A*_fabricated_ (if the experimental conditions allow it) as a recommended control experiment. While smaller areas are useful for screening new materials, reporting at least one baseline curves at a 1:1 ratio ensures realistic scalability and prevents over-optimistic kinetic interpretations.

This reporting procedure would provide a more transparent assessment of area dependent transport limitations and the true scalability of new photoactive materials. Ultimately, this study provides guidelines to avoid overestimation of performance and contributes to the reliable evaluation of water oxidation materials for solar fuel applications.

## Supplementary Material



## Notes

Characterization of the BiVO_4_ photoanodes, including FE-SEM, EDS, UV–vis spectroscopy, Tauc analysis and IPCE measurements; additional information on the photoelectrochemical characterization, including experimental setup, front and back illumination measurements, impedance spectroscopy, and infrared-chopped chronoamperometry; summary of reported BiVO_4_ photoanode performances and exposed-to-fabricated area ratios, together with the methodology and parameters obtained from the simultaneous fitting of IS, IMPS and IMVS measurements under front and back illumination, as well as Raman spectroscopy results (PDF)
