# Supplementary material for: Understanding the Exposed Area Dependent Photoelectrochemical Performance of BiVO4 Photoanodes
Source: ACS Meas Sci Au. 2026 Sep 16;6(5):1898–906. doi: 10.1021/acsmeasuresciau.6c00248 (PMC13693429; doi:10.1021/acsmeasuresciau.6c00248)
Supplement: Supplementary file 1 [file tg6c00248_si_001.pdf]

Supporting Information

Understanding the exposed area dependent photoelectrochemical performance of $BiVO_4$ photoanodes

Christian Robles,[1] Ana Gutiérrez-Blanco,[1,] Laura Montañés,[1] Agustin O. Alvarez,[1,2]*

Camilo A. Mesa,[1,] Francisco Fabregat-Santiago,[1] Sixto Giménez,[1,*]*

[1] Institute of Advanced Materials, Universitat Jaume I, Avinguda de Vicent Sos Baynat, s/n, 12006 Castelló de la Plana, Spain

[2] AMOLF, Science Park 104, 1098 XG Amsterdam, the Netherlands

* Corresponding authors: angutier@uji.es, camilo.mesa@icn2.cat, sjulia@uji.es

Synthesis of $BiVO_4$ photoanodes

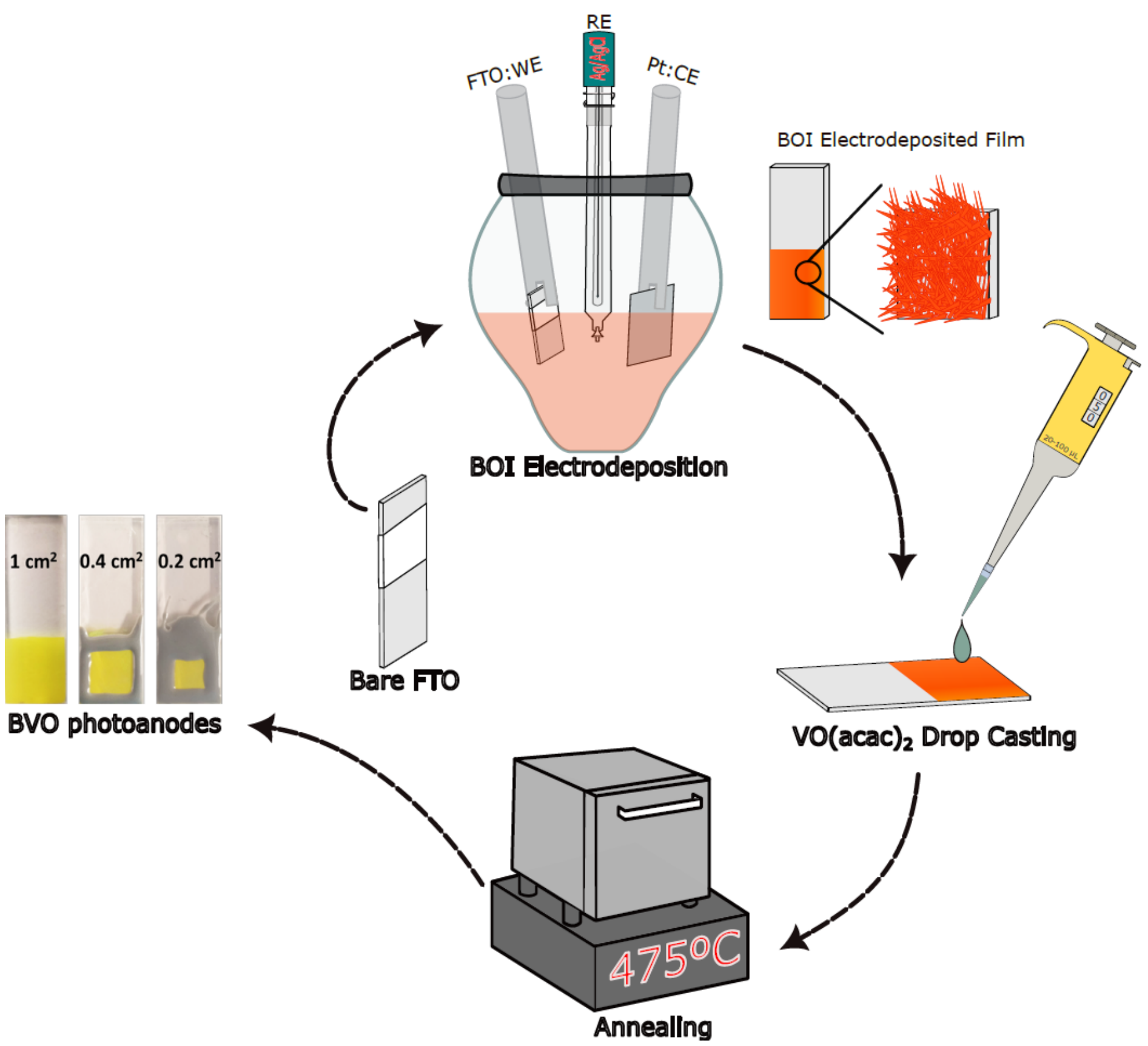

Figure S1. Scheme of the electrochemical synthesis of $BiVO_4$ photoanodes.

Morphological and Photoelectrochemical Characterization

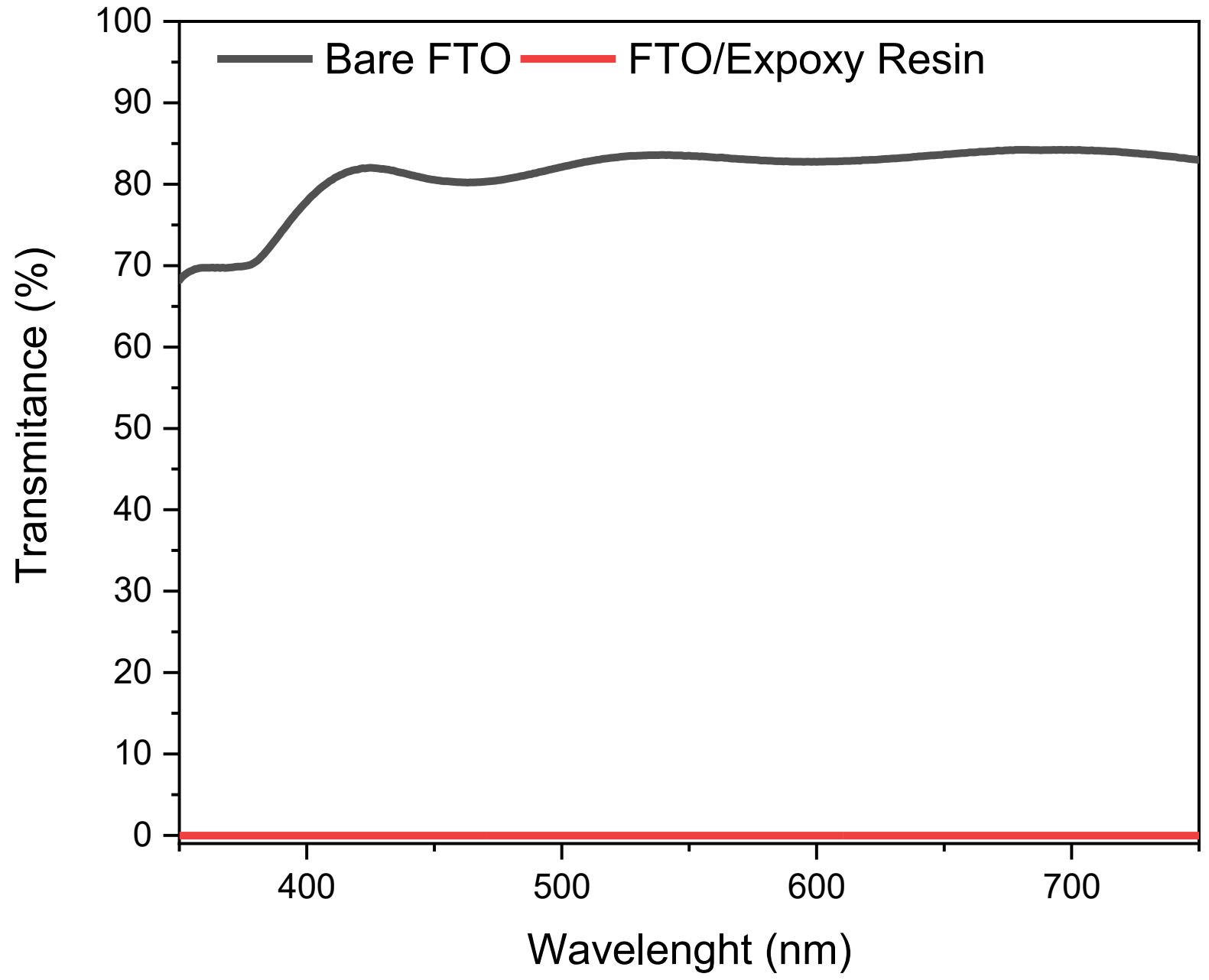

Figure S2. Transmittance spectra of a bare FTO substrate and an FTO substrate coated with the epoxy resin.

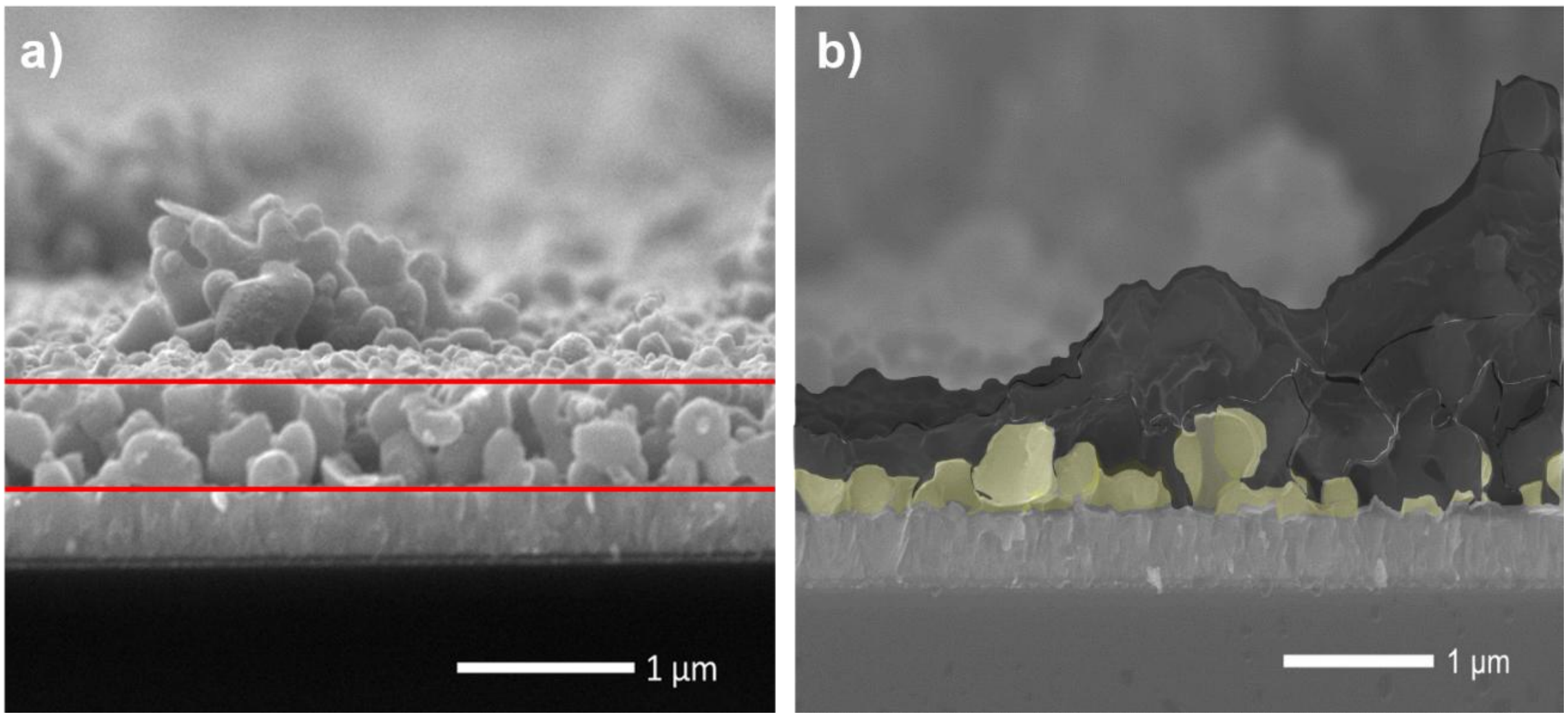

Figure S3. Cross-section Scanning Electron Microscopy (SEM) image of a) $BiVO_4$ photoanode exhibiting a thickness of ~0.6 µm and **b)** $BiVO_4$ photoanode (yellow colour) with the opaque epoxy resin mask (dark grey colour).

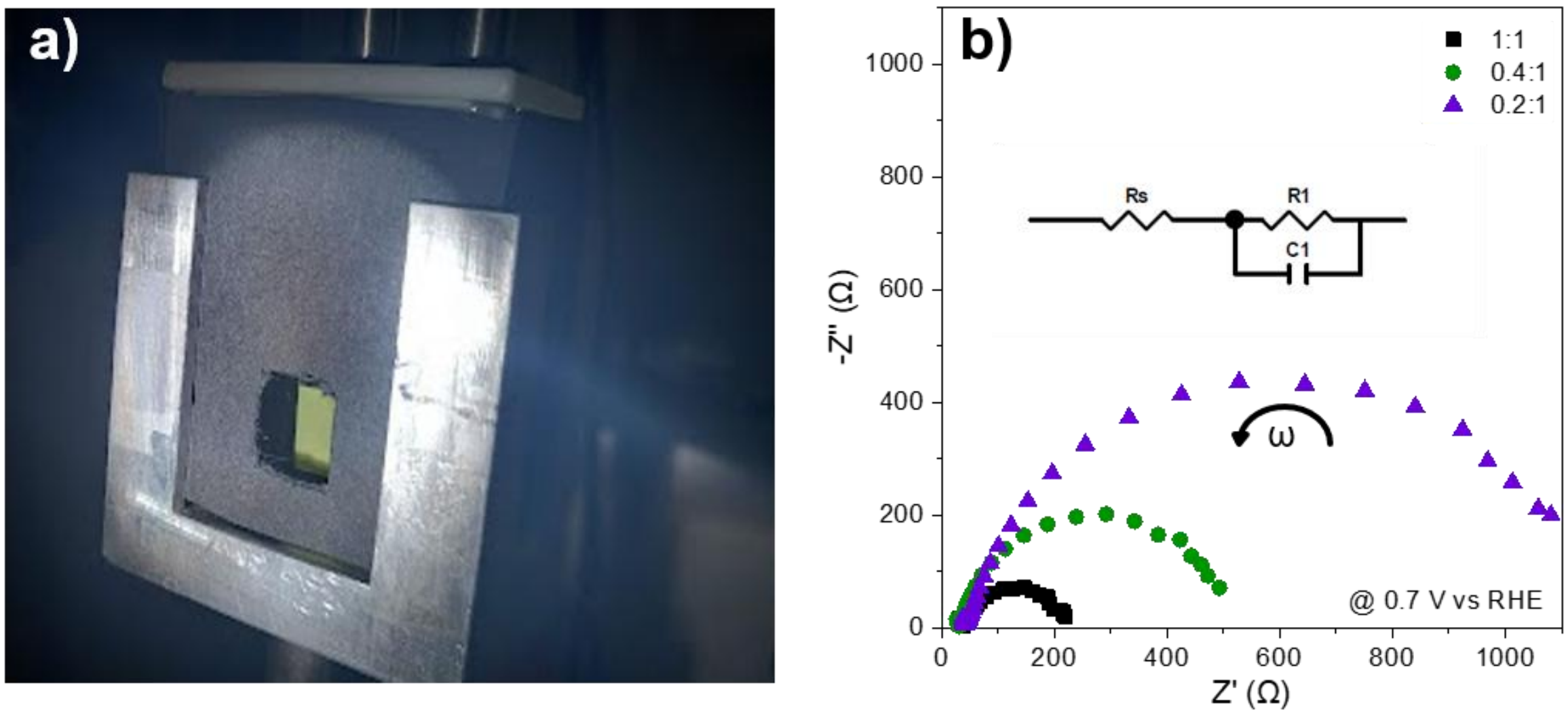

Figure S4. a) Photograph of the PEC cell used for PEC measurements, b) Representative Nyquist plot and equivalent circuit model employed for fitting the results from IS. The direction of increasing frequency (ω) is indicated. The last low frequency points deviating from the circular shape were not included for the fitting process.

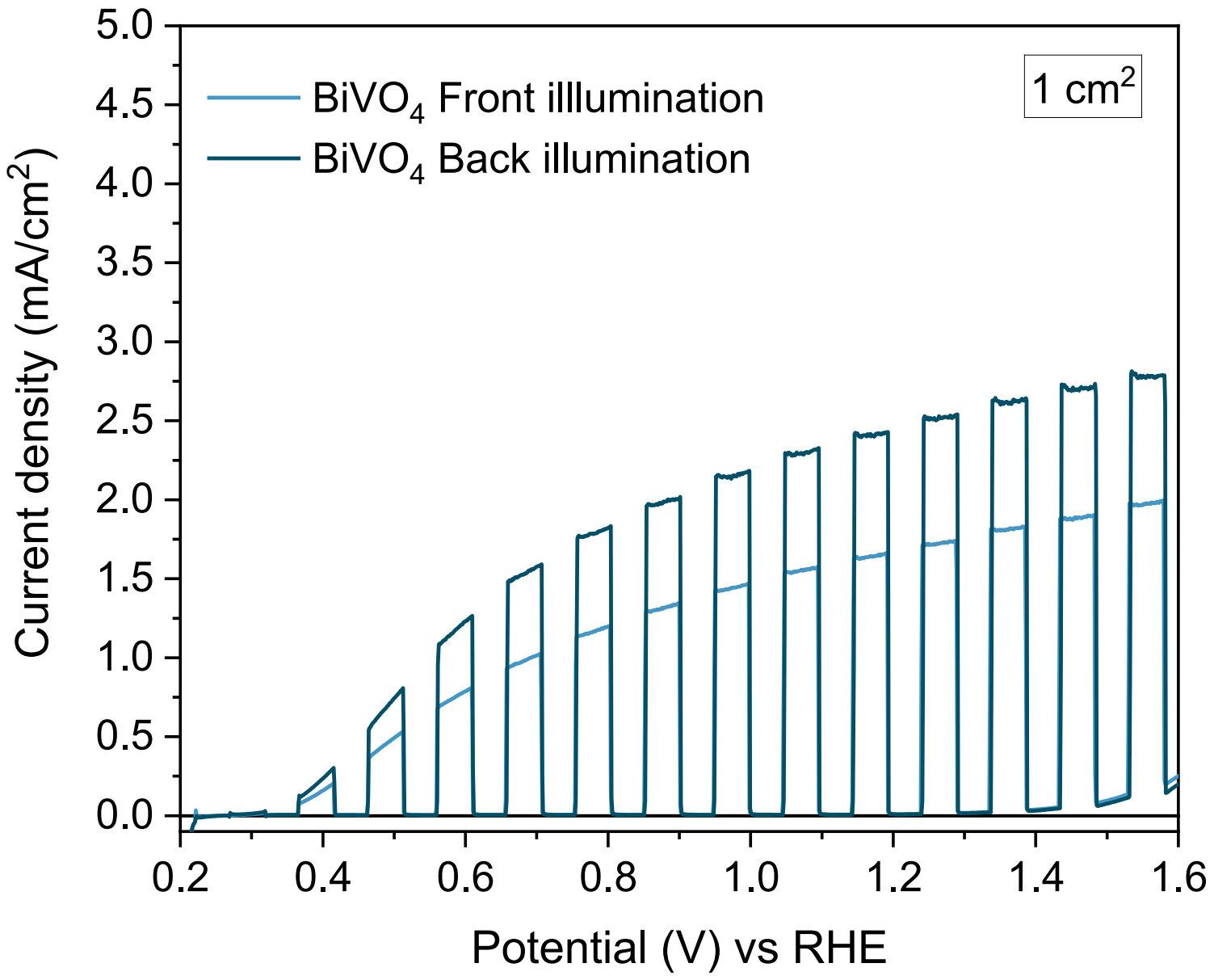

Figure S5. Current–voltage (J–V) plots measured by front illumination (light blue) and back illumination (dark blue) in 0.1 M KPi with 0.1 M Na_2SO_3.

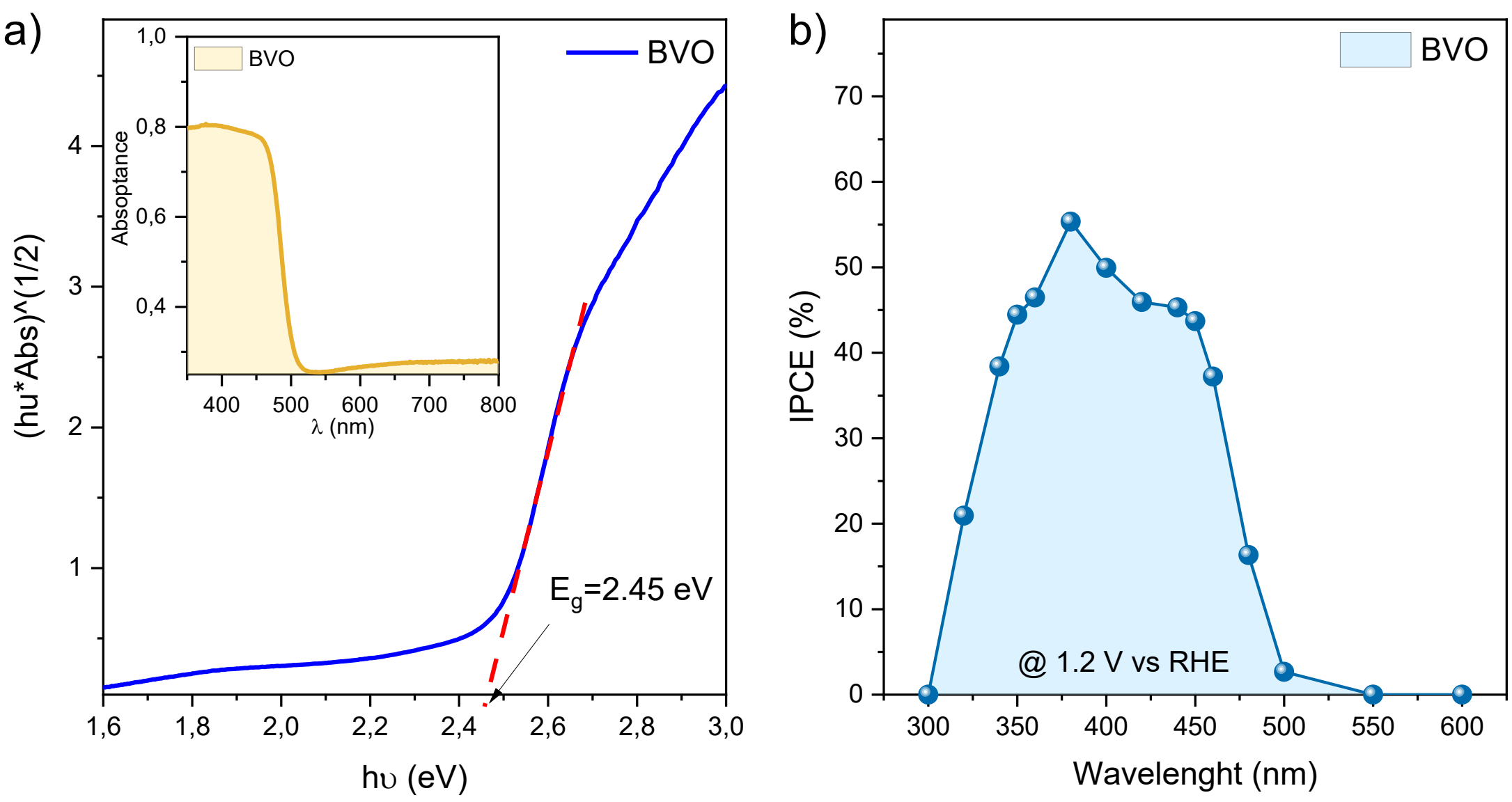

Figure S6. a) Tauc plot for the determination of the indirect bandgap, inset: absorptance spectrum of the $BiVO_4$ film for the calculation of the Tauc plot and **b)** Incident-photon to current efficiency (IPCE) of a $BiVO_4$ photoanode at 1.23 V *vs.* RHE, under back illumination measured at 0.1 M KPi with 0.1 M Na_2SO_3.

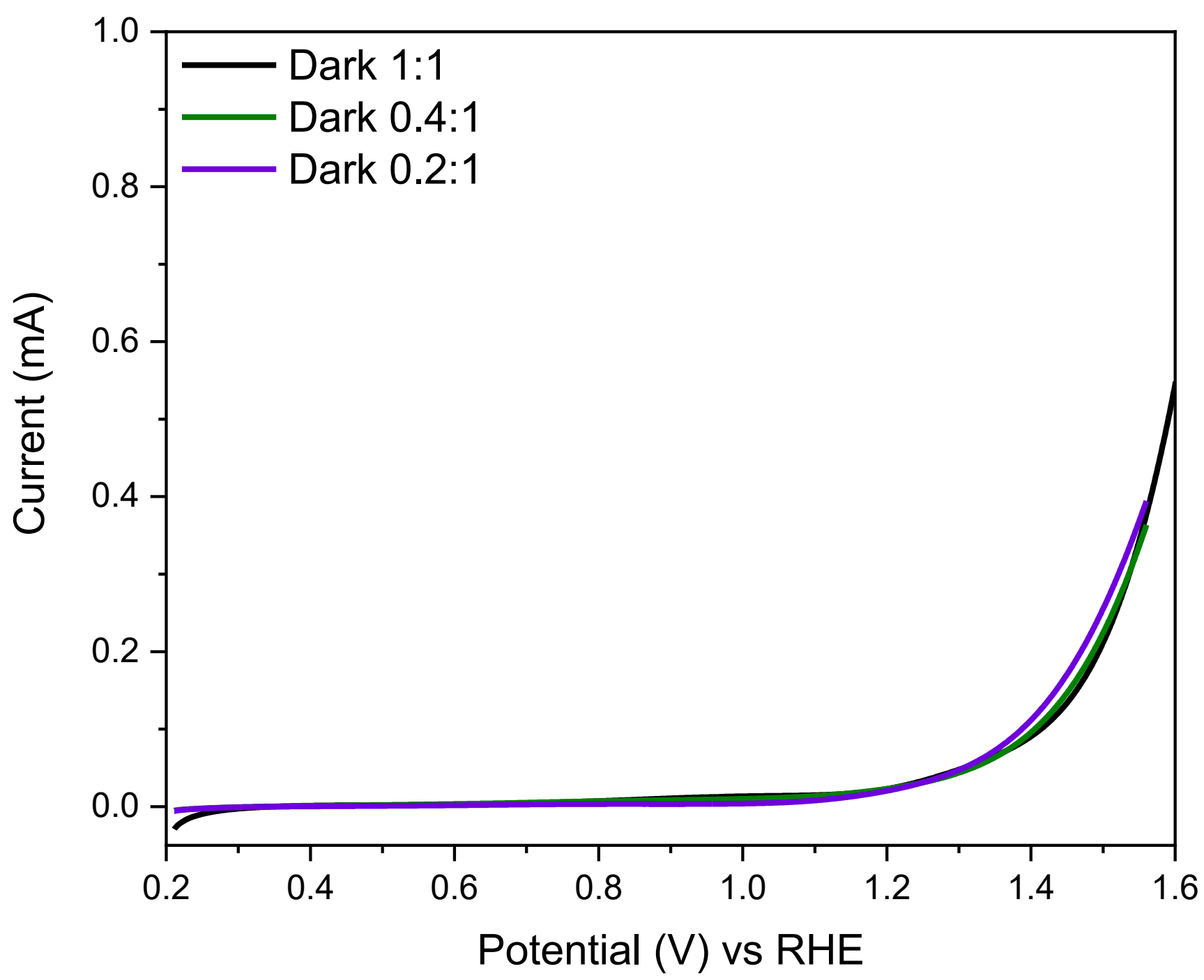

Figure S7. Dark currents from Figure 2a without area normalization.

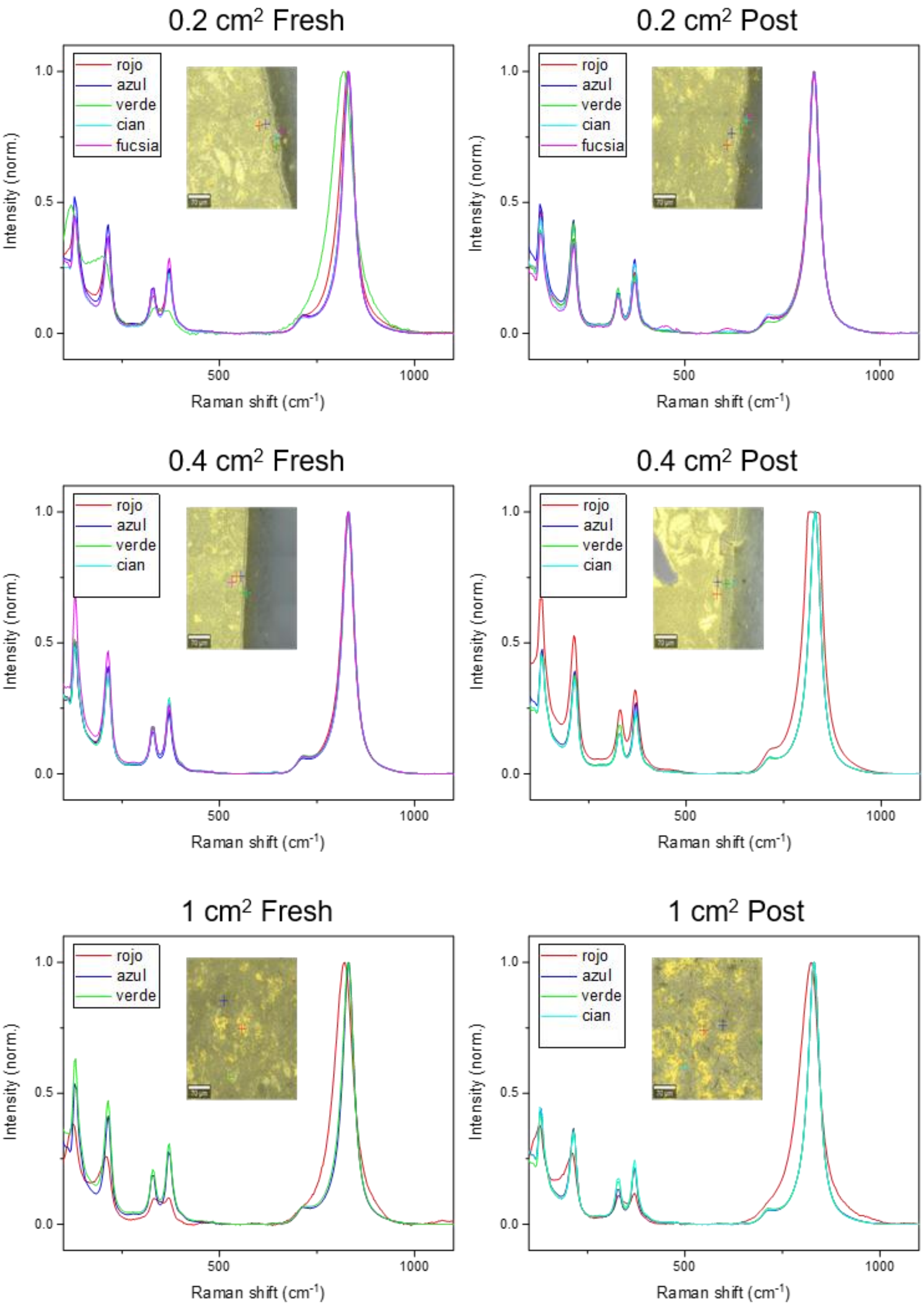

Figure S8. Raman spectra for the three different exposed areas before and after measuring. Colours represent different spots measured in the same sample.

Table S1. Summary of photoelectrochemical performance for $BiVO_4$ based photoanodes reported in the literature under 1 sun illumination: Photoactive exposed area (PA), fabricated area (FA), exposed to fabricated area ratio (EFAR), photocurrent density at 1.23 V vs RHE (J_{ph}), hole scavenger or catalyst (HS or Cat), illumination direction (ID: front (F) or back (B)).

PA (cm^2)	FA (cm^2)	EFAR	J_{ph} ($mA \cdot cm^{-2}$)	HS/Cat	ID	Ref
1	3	0.3	2.91	HS	B	1
			5.1	Cat		
0.24	50	0.0048	3.3	HS	B	2
50		1	1.2	HS		
50			1.5	Cat		
0.24	1	0.24	3.5	HS	F	3
			6.1	Cat		
0.2	1.3	0.15	3.2	Cat	B	4
			4.2	Cat		
			3.3	Cat		
			3.6	Cat		
			3.3	HS		
0.24	5	0.05	5	HS	F	5
1	1	1	1.72	HS	F	6
1	1	1	5.89	Cat	F	7
0.8	0.8	1	1.3	HS	B	8
0.13	2	0.06	5.4	HS	B	9
			5.5	Cat		
1	1	1	2.9	Cat	B	10
0.1	1	0.1	3.7	Cat	B	11
			4.8	Cat		
			5	HS		
0.25	1.13	0.024	4	HS	F	12
0.05	0.12	0.41	3.6	HS	B	13
0.5	4	0.125	2.2	HS	B	14
0.26	7	0.037	5.15	HS	F	15
0.25	1	0.25	5.82	HS	F	16
			2.5	HS		
50	50	1	0.5	HS	B	17
3.11	25	0.12	4.27	Cat	F	18
1	1	1	0.89	HS	B	19
25	25		0.67	HS	B	
1	1	1	1.58	Cat	B	20
0.2	1	0.2	2.92	HS	B	This work
0.4		0.4	2.89			
1		1	2.25			

Table S2. Parameters extracted from the simultaneous fitting of the IS, IMPS and IMVS spectra for front and back illumination.

Sample	1:1		0.4:1		0.2:1	
**Illumination Side**	**Front**	**Back**	**Front**	**Back**	**Front**	**Back**
**Diffusion Species**	**Electrons**	**Holes**	**Electrons**	**Holes**	**Electrons**	**Holes**
R_s [Ω cm^2]	35.9	36.7	21.6	22	15.7	12.8
R_{tr} [Ω cm^2]	23	25	13	8.1	5	3.8
R_{ct} [Ω cm^2]	1283	984	716	385	536	281
$\Delta R_{ct}/R_{ct}$ [%]	0	0	79	156	139	250
C_μ [μF cm^{-2}]	15	15	16	20	25	30
C_μ / $C_{\mu\ 1cm2}$ [%]	100	100	107	133	167	200
ω_{rec} [rad/s]	53	69	89	131	75	119
τ_{rec} [ms]	18.9	14.4	11.2	7.7	13.4	8.4
ω_D [rad/s]	2933	2726	4869	6169	7437	8891
L/d	7.4	6.3	7.4	6.9	10	8.6
D [10^{-6} cm^2 s^{-1}]	7.33	6.81	12.2	15.4	18.6	22.2
L [nm]	3719	3137	3699	3436	4992	4313
μ [10^{-4} cm^2 V^{-1} s^{-1}]	2.84	2.63	4.71	5.96	7.19	8.60
a [%]	98.2	98.2	98.2	98.2	98.2	98.2
η_{opt} [%]	79.9	75.1	79.9	75.1	79.9	75.1
η_{col} [%]	96	95	96	94	97	95
η_{sep} [%]	5.7	8	5.7	8	5.7	8
η_{geo} [%]	100	100	84.2	156.2	156.1	222.5
η_{sep}' [%]	5,7	8,0	4,8	12,5	8,9	17,8
$IQE_{PV\text{-}diff}$ [%]	5.5	7.6	4.6	11.7	8.6	16.9
$EQE_{PV\text{-}diff}$[%]	4.3	5.6	3.6	8.6	6.7	12.5

Table S3. Parameters extracted from the simultaneous fitting of the IS, IMPS and IMVS spectra for front and back illumination, without normalizing to the areas.

Sample code	1:1		0.4:1		0.2:1	
**Illumination Side**	**Front**	**Back**	**Front**	**Back**	**Front**	**Back**
**Diffusion Species**	**Electrons**	**Holes**	**Electrons**	**Holes**	**Electrons**	**Holes**
R_s [Ω]	35.9	36.7	54	55	78.5	64
R_{tr} [Ω]	23	25	32.5	20.25	25	19
R_{rec} [Ω]	1283	984	1790	962.5	2680	1405
C [μF]	15	15	6.4	8	5	6

References

(1) Mane, P.; Bagal, I. V.; Bae, H.; Burungale, V.; Seong, C.; Ryu, S. W.; Ha, J. S. Bifacial Modulation of Carrier Transport in BiVO4 Photoanode for Stable Photoelectrochemical Water Splitting via Interface Engineering. *Adv. Sustain. Syst.* **2022**, 6, 2200014.

(2) Ahmet, I. Y.; Ma, Y.; Jang, J. W.; Henschel, T.; Stannowski, B.; Lopes, T.; Vilanova, A.; Mendes, A.; Abdi, F. F.; Van De Krol, R. Demonstration of a 50 Cm2 BiVO4 Tandem Photoelectrochemical-Photovoltaic Water Splitting Device. *Sustain. Energy Fuels* **2019**, 3, 2366–2379.

(3) Han, H. S.; Shin, S.; Kim, D. H.; Park, I. J.; Kim, J. S.; Huang, P. S.; Lee, J. K.; Cho, I. S.; Zheng, X. Boosting the Solar Water Oxidation Performance of a BiVO4 Photoanode by Crystallographic Orientation Control. *Energy Environ. Sci.* **2018**, 11, 1299–1306.

(4) Tae Woo, K.;K Choi, K. Nanoporous BiVO4 Photoanodes with Dual-Layer Oxygen Evolution Catalysts for Solar Water Splitting. *Science.* **2014**, 343, 990–994.

(5) Toma, F. M.; Cooper, J. K.; Kunzelmann, V.; McDowell, M. T.; Yu, J.; Larson, D. M.; Borys, N. J.; Abelyan, C.; Beeman, J. W.; Yu, K. M.; Yang, J.; Chen, L.; Shaner, M. R.; Spurgeon, J.; Houle, F. A.; Persson, K. A.; Sharp, I. D. Mechanistic Insights into Chemical and Photochemical Transformations of Bismuth Vanadate Photoanodes. *Nature Communications* **2016**, 7, 1–11.

(6) Yang, L.; Xiong, Y.; Guo, W.; Guo, J.; Gao, D.; Zhang, Y.; Xiao, P. Mo6+ Doped BiVO4 with Improved Charge Separation and Oxidation Kinetics for Photoelectrochemical Water Splitting. *Electrochim. Acta* **2017**, 256, 268–277.

(7) Nikandish, M.; Taherinia, D.; Ghasemian, S. Electrodeposited CoSe2 Supported on BiVO4 as an Efficient Photoanode for the Enhanced Photoelectrochemical Water Oxidation. *Sustain. Energy Fuels* **2025**, 9, 2149–2158.

(8) Okunaka, S.; Hitomi, Y.; Tokudome, H. Green Fabrication of Nanoporous BiVO4 Films on ITO Substrates for Photoelectrochemical Water-Oxidation. *RSC Adv.* **2018**, 8, 31575–31580.

(9) Wang, S.; He, T.; Chen, P.; Du, A.; Ostrikov, K.; Huang, W.; Wang, L. In Situ Formation of Oxygen Vacancies Achieving Near-Complete Charge Separation in Planar BiVO4 Photoanodes. *Adv. Mater.* **2020**, 32, 2001385.

(10) Salih, A. K.; Khan, A. Z.; Drmosh, Q.; Kandiel, T.; Qamar, M.; Jahangir, T.; Ton-That, C.; Yamani, Z. Nanostructured BiVO4 Photoanodes Fabricated by Vanadium-Infused Interaction for Efficient Solar Water Splitting. *ACS Appl. Nano Mater.* **2024**, 7, 14115–14122.

(11) Kim, T. W.; Ping, Y.; Galli, G. A.; Choi, K. S. Simultaneous Enhancements in Photon Absorption and Charge Transport of Bismuth Vanadate Photoanodes for Solar Water Splitting. *Nature Communications* **2015**, 6, 8769.

(12) Milbrat, A.; Vijselaar, W.; Guo, Y.; Mei, B.; Huskens, J.; Mul, G. Integration of Molybdenum-Doped, Hydrogen-Annealed BiVO 4 with Silicon Microwires for Photoelectrochemical Applications. *ACS Sustain. Chem. Eng.* **2019**, 7, 5034–5044.

(13) Kang, D.; Park, Y.; Hill, J. C.; Choi, K. S. Preparation of Bi-Based Ternary Oxide Photoanodes BiVO4, Bi2WO6, and Bi2Mo3O12 Using Dendritic Bi Metal Electrodes. *Journal of Physical Chemistry Letters* **2014**, 5, 2994–2999.

(14) Gacha, V.; Ros, C.; Garcia, X.; Llorca, J.; Martorell, J.; Raptis, D. Enhancing Charge Extraction in BiVO4 Photoanodes by ZrCl4 Treatment of SnO2 Hole-Blocking Layers. *Appl. Mater. Today* **2024**, 40, 102415.

(15) Jian, J.; Xu, Y.; Yang, X.; Liu, W.; Fu, M.; Yu, H.; Xu, F.; Feng, F.; Jia, L.; Friedrich, D.; van de Krol, R.; Wang, H. Embedding Laser Generated Nanocrystals in BiVO4 Photoanode for Efficient Photoelectrochemical Water Splitting. *Nature Communications* **2019**, 10, 2609.

(16) Qiu, Y.; Liu, W.; Chen, W.; Zhou, G.; Hsu, P. C.; Zhang, R.; Liang, Z.; Fan, S.; Zhang, Y.; Cui, Y. Efficient Solar-Driven Water Splitting by Nanocone BiVO4-Perovskite Tandem Cells. *Sci. Adv.* **2016**, 2, e1501764.

(17) Robles, C.; Montañés, L.; Mesa, C. A.; Iglesias, D.; Rabelo, H.; Spadaro, M. C.; Arbiol, J.; Redondo, J.; Schiller, F.; Barja, S.; Julián-López, B.; Gutiérrez-Blanco, A.; Sans, V.; Giménez, S. Continuous-Flow Synthesis of BiVO4 Nanoparticles: From Laboratory Scale to Practical Systems. *ChemSusChem* **2025**, 18, e202402583.

(18) Xu, Z.; Chen, L.; Brabec, C. J.; Guo, F. All Printed Photoanode/Photovoltaic Mini-Module for Water Splitting. *Small Methods* **2023**, 7, 2300619.

(19) Murugan, C.; Mary, A. S.; Pandikumar, A. Fabrication and Performance Validation of BiVO4 Photoanode-Based Photoelectrochemical Cells with Different Sizes and Reducing the Photocurrent Density Loss with Different Patterns. *Ind. Eng. Chem. Res.* **2024**, 63, 4329–4337.

(20) Yao, X.; Wang, D.; Zhao, X.; Ma, S.; Bassi, P. S.; Yang, G.; Chen, W.; Chen, Z.; Sritharan, T. Scale-Up of BiVO4 Photoanode for Water Splitting in a Photoelectrochemical Cell: Issues and Challenges. *Energy Technology* **2018**, 6, 100–109.
